# Assessing the Microbial Community and Functional Genes in a Vertical Soil Profile with Long-Term Arsenic Contamination

**DOI:** 10.1371/journal.pone.0050507

**Published:** 2012-11-30

**Authors:** Jinbo Xiong, Zhili He, Joy D. Van Nostrand, Guosheng Luo, Shuxin Tu, Jizhong Zhou, Gejiao Wang

**Affiliations:** 1 State Key Laboratory of Agricultural Microbiology, College of Life Science and Technology, Huazhong Agricultural University, Wuhan, China; 2 Institute for Environmental Genomics, Department of Botany and Microbiology, University of Oklahoma, Norman, Oklahoma, United States of America; 3 College of Resources and Environment, Huazhong Agricultural University, Wuhan, China; Argonne National Laboratory, United States of America

## Abstract

Arsenic (As) contamination in soil and groundwater has become a serious problem to public health. To examine how microbial communities and functional genes respond to long-term arsenic contamination in vertical soil profile, soil samples were collected from the surface to the depth of 4 m (with an interval of 1 m) after 16-year arsenic downward infiltration. Integrating BioLog and functional gene microarray (GeoChip 3.0) technologies, we showed that microbial metabolic potential and diversity substantially decreased, and community structure was markedly distinct along the depth. Variations in microbial community functional genes, including genes responsible for As resistance, carbon and nitrogen cycling, phosphorus utilization and cytochrome *c* oxidases were detected. In particular, changes in community structures and activities were correlated with the biogeochemical features along the vertical soil profile when using the *rbcL* and *nifH* genes as biomarkers, evident for a gradual transition from aerobic to anaerobic lifestyles. The C/N showed marginally significant correlations with arsenic resistance (*p* = 0.069) and carbon cycling genes (*p = *0.073), and significant correlation with nitrogen fixation genes (*p = *0.024). The combination of C/N, NO_3_^−^ and P showed the highest correlation (*r* = 0.779, *p* = 0.062) with the microbial community structure. Contradict to our hypotheses, a long-term arsenic downward infiltration was not the primary factor, while the spatial isolation and nutrient availability were the key forces in shaping the community structure. This study provides new insights about the heterogeneity of microbial community metabolic potential and future biodiversity preservation for arsenic bioremediation management.

## Introduction

Arsenic (As) contamination of soil and water has become a serious problem due to its widespread distribution and high toxicity [1]. Natural As-contaminated groundwater has been reported all over the world [2], [3], [4], and this contamination has become a major threat to public health. Chronic drinking of As-contaminated groundwater has caused endemic arsenicosis [5], and As-contaminated soils have resulted in an accumulation of As in rice grains [6]. In order to remediate the As-contaminated soil, *Pteris vittata*, an As hyperaccumulating plant, was planted in area of Chenzhou, China, where the soil was contaminated by an adjacent arsenic smelting. The aboveground biomass of *P. vittata* was harvested each year, and the roots were left in the soil, which re-grew in the following year. After continual phytoremediation for 6 years, the soil arsenic level was low enough for plant cultivation [7]. Paiticularlly, we found that the rhizosphere of *P. vittata* harbored special microbial structures and genes [7]. However, the downward infiltration of arsenic has caused serious groundwater contamination in the adjacent untreated area, while the response of underground microbial communities and their interactions during this process are largely unknown.

Microorganisms play a detrimental role in the As geochemical cycle, mainly driving As redox and methylation [8], [9], [10], [11], which were previously thought to be a cellular detoxification strategy. Recently, a photosynthetic bacterium showed the ability to use As(III) as sole electron donor to fix CO_2_ [12], implying that microbial metabolism of As is involved in energy source flow. Many microorganisms that interact with and transform inorganic As species have been identified [11], [13], [14]. In particular, As-resistant bacteria had been isolated from underground sediments at a depth of 41 m, and their distribution correlated with geological factors across soil depths [10]. Although soil microorganisms play a critical role in the mobilization of As in the subsurface aquifer and are important for the maintenance of groundwater quality [15], [16], little is known about the factors that generate the microbial community structure and activity.

Numerous studies have shown that oxygen concentration, nutrient bioavailability and water content influence microbial community diversity patterns along soil depths [17], [18], [19], [20]. Particularly, in some *Thiomonas* strains, genes related to inorganic carbon assimilation and metabolism are induced by arsenic and linked with arsenic metabolism [21], indicating a direct correlation between the microbial arsenic metabolism and soil carbon availability. Recently, there is ample evidence that arsenic contamination exerted strong selective pressures on microbial communities [7], [11]. However, the knowledge of the linkage between key microbial functional genes (pathways) and vertical soil heterogeneities is limited, especially for those factors coupled with arsenic contamination. Understanding the complex relationships between geochemical factors and microbial functional genes involved in arsenic transformation is crucial for biodiversity protection and management of desired microbial populations for arsenic bioremediation [22].

We hypothesized that arsenic infiltration, spatial isolation and vertical heterogeneity would allow for the differentiation of microbial community structures and functional processes, consequently altering the toxicity and mobility of arsenic to groundwater. To test this idea, samples were collected from two soil vertical columns (from the surface down to 4 m) after 16-year arsenic contamination, in Chenzhuo, China. The microbial metabolic potential and community structure were simultaneously characterized using BioLog system and functional gene microarray (GeoChip 3.0) [23]. We were particularly interested in determining what functional genes and geochemical factors played most important roles in the physical and biogeochemical processes in this system. Our results revealed that the microbial diversity, structure, metabolic potential, and key functional genes varied greatly along the vertical soil profile, and that the nutrient availability and spatial isolation could be key factors in shaping the soil microbial community structure.

## Materials and Methods

### Ethics Statement

No specific permits were required for the described field studies. No specific permissions were required for these locations/activities because sample collection did not involve endangered or protected species or privately owned location.

### Site Description and Soil Sample Collection

Soil samples were collected from an As-contaminated area that had been seriously contaminated for 7 years (from 1992 to 1999; in 1999, the factory was closed by the local government) by the waterfall waste from an adjacent As smelting factory, located in Dengjiatang village, Chenzhuo city, Hunan province, China (25°48′N, 113°02′E). In 2008, soil samples were collected from two vertical soil columns with 5 depths (from the surface to a depth of 4 m with an interval of 1 m) after 16-year arsenic contamination (from 1992 to 2008). At each depth, samples were mixed thoroughly and triplicate divided (pseudo-biological replicates). Thus, six subsamples from each depth were used for the Biolog and GeoChip analyses.

### Utilization of Sole Carbon Substrates

The microbial community carbon utilization ability was immediately assayed (within one month) via the EcoPlate™ according to the manufacturer’s instructions. Briefly, five grams soil from each sample was added to 45 ml ddH_2_O and incubated at 4°C with 200 rpm for 45 min and was then left standing for 30 min. The samples were serially diluted to 10^−3^ based on a pilot experiment, 100 µl of each sample was inoculated into each well, and the samples were incubated at 25°C for 168 hrs within an OminLog System (BioLog Inc., Hayward, CA). The average metabolic response (AMR) of the soil samples was calculated as the average of the mean difference between the O.D. of the carbon source containing wells and the control wells [24].

### Genomic DNA Extraction and Purification

Total community DNA was extracted with 5 g soil using a freeze-grinding method and SDS for cell lysis as described previously [25]. The crude DNA was purified via low melting agarose gel electrophoresis, followed by phenol–chloroform–butanol extraction. DNA was quantified with a PicoGreen kit (Invitrogen, Carlsbad, CA, USA). Purified DNA was stored at −20°C until use.

### GeoChip Hybridization

An aliquot (100 ng) of purified DNA from each sample was amplified using the TempliPhi kit (Amersham Biosciences, Piscataway, NJ) with a modified buffer containing single-stranded-binding protein (200 ng/ml) and spermidine (0.04 mM) to increase the sensitivity of amplification, and each sample was incubated at 30°C for 6 hrs. The amplified products (2 µg) were fluorescently labeled and purified with the Wizard DNA Clean-up System (Promega, Madison, WI). The labeled DNA was dried and resuspended in 50 µl of hybridization solution containing 50% formamide, 5×SSC, 0.1% SDS, and 0.1 mg/ml herring sperm DNA. Hybridization of the GeoChip 3.0 was performed on an MAUI Hybridization Station (BioMicro®, Salt Lake City, UT) at 42°C for 12 hrs. Hybridized slides were semi-automatically washed with a MAUI wash system (BioMicro®) and then scanned using a ScanArray 5000® Microarray Analysis System (PerkinElmer, Wellesley, MA).

### GeoChip Data Analysis

The signal intensities of each spot were measured with the ImaGene™ 6.1 (Biodiscovery Inc., Los Angeles, CA) instrument. Only the spots that were automatically scored as positive in the raw output data were used for further analysis. The signal intensities used for the final analysis were divided by the mean of each sample. Spots with signal-to-noise ratio (SNR) <2.0 [SNR = (signal intensity - background mean)/background standard deviation] were removed. A gene was considered positive when a positive hybridization signal was obtained at least twice (≥2) out of the six biological replicates tested for each depth.

### Statistical Analysis

Detrended correspondence analysis (DCA) was performed to evaluate the differences in the microbial communities [26]. One-way ANOVA procedures with Kutey test were performed to detect the significant differences of the functional genes among samples at different depths [27]. Phylogenetic tree construction used the neighbor-joining distance method. Mantel tests were used to examine the correlations between soil chemical concentrations and functional gene abundances. Cluster analysis was performed using the pairwise average linkage hierarchical clustering algorithm and visualized by Treeview software (http://rana.stanford.edu).

## Results and Discussion

### Physical and Chemical Characterization of the Soil Samples

Soil textures varied considerably with the soil depths (Table 1). Soluble As concentrations significantly exceeded the limit set by the Standard for Environmental Quality of Soils (GB15618-1995). In general, the nutrient availabilities decreased, while the moisture increased along the vertical soil profile (Table 1), indicating that the deeper layers were more nutrient poor, which may be limited in electron acceptors.

**Table 1:** Selected physical and chemical properties of the soils*^a.^*

Depths	Texture	Moisture	pH	TC	TN	C/N	As	NO_3_^−^	P	Fe	S
				g/kg			mg/kg				
0-m	silty clay	20%	8.1	26.40	1.68	15.75	75.6	25.59	426	41.4	717
1-m	loam clay	19%	6.9	15.64	1.49	10.48	72.7	16.23	245	35.0	470
2-m	silty clay loam	27%	6.9	5.49	1.34	4.10	66.0	4.78	206	53.5	275
3-m	loam clay	33%	7.2	3.54	1.20	2.95	65.5	9.15	192	63.4	453
4-m	clay	28%	6.5	5.20	1.04	5.00	65.0	12.36	236	50.4	407

a average values of 6 subsamples at each depth.

pH, 1∶2.5 soil-H_2_O suspension; As, 0.05 M (NH_4_)_2_SO_4_ soluble concentration.

A depth of 0 m represents soil of 0.00–0.10 m underground; 1, 2, 3 and 4 m represent ±0.05 m at each depth underground. TC, total carbon; TN, total nitrogen.

### Utilization of Sole Carbon Sources among the Five-depth Soil Samples

**Figure 1:**
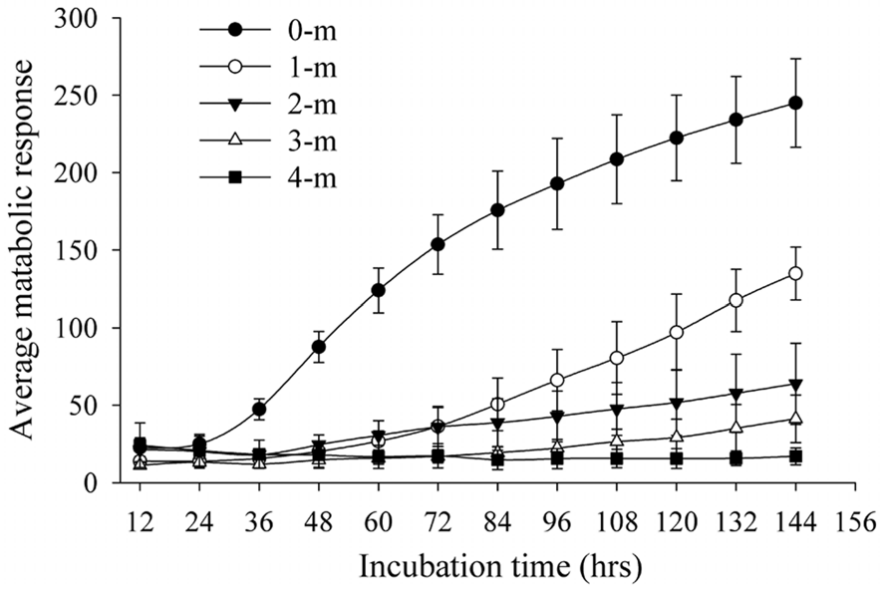
Average metabolic response (AMR) of the soil samples measured by the BioLog system, error bar indicates ± 1 SE (standard error, *N = *6) of the six biological replicates within each depth.

The average metabolic response (AMR) values decreased strongly with the soil depth, and the carbon metabolic diversity was much higher in the surface (0-m) samples than those of the other samples (Fig. 1), indicating that the carbon metabolic potential was negatively correlated with depth. A previous study reported that the surface soil harbored a higher proportion of physiologically or phylogenetically pre-adapted inhabitants for rapid metabolism of labile carbon substrates [18]. Consistently, a much higher metabolic potential was observed in the surface soil samples, while the 4-m samples did not display ability or need much more time to resuscitate (dormant microorganisms) to metabolize any of the carbon sources in the EcoPlate (Fig. 1), which indicated that its metabolic potential was distinct from other soil depths. It should be noted that the Biolog system detects the sole carbon utilization ability under aerobic conditions, while the underground (1-, 2-, 3- and 4-m) habitants may have adapted to anaerobic respiration conditions. Such differences may be due to the variation of metabolic substrates and/or soil oxygen availability from depths, as previous studies have reported [17], [18].

### Microbial Functional Gene Diversity and Distribution

After hybridization, a total of 6,004 functional genes were detected in at least one depth of the soil samples. Among these, 2,848 (accounting for 47.4%) genes were unique and only detected at a single depth. In contrast, fewer genes (1,344, or 22.4%) were shared among all five soil depth samples, indicating that the microbial community compositions were distinct from each other.

A substantial number of genes (2,489) were detected at the surface soil (0-m), while the number of genes was significantly lower in underground soils, varied from 882 to 1,522 (Table S1). Previous studies have shown that microbial diversity substantially declined in deeper soil samples [13], [19]. Consistently, both Simpson reciprocal (*1/D*) and Shannon–Weaver (*H′*) diversity indices showed much higher functional gene diversities in the surface samples, e.g., *1*/*D* was 1433.0 at the surface soils, and 485.5 to 840.0 for other soil depths (Table S1). Greater nutrient heterogeneity at the surface soil maintained a high-level microbial diversity [19].

**Figure 2:**
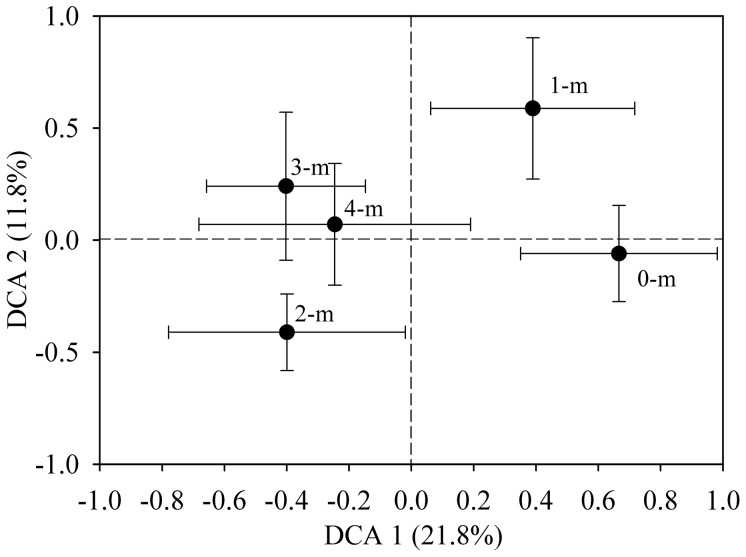
Detrended correspondence analysis (DCA) of detected functional genes at different soil depths. Error bars indicate ± 1 SE (*N = *6).

Detrended correspondence analysis (DCA) of all detected genes was also used to examine overall functional structure changes in the microbial communities, which showed a substantial level of differentiation in microbial community structure with the depth, and explained 33.6% of the total variance (Fig. 2). The deeper layer samples clustered more tightly than the upper layer samples, and the microbial community structure of 3-m and 4-m samples could not separate well (Fig. 2). Thus, the spatial isolation could be a key factor in shaping the bacterial community structures across the vertical profile.

Microbial composition and relative abundance were compared among the five depths. Based on the abundance of functional genes, α-, β- and γ-*Proteobacteria* were the dominant groups across the samples, representing 20.1–25.7%, 11.8–17.3% and 18.3–21.3%, respectively. Furthermore, the 0-m samples harbored higher abundances of α- and β-*Proteobacteria*, while samples from the underground soil had higher abundances of fungi and γ-*Proteobacteria* (data not shown). Similarly, a survey of flooded paddy soils detected a predominance of α- and β-*Proteobacteria* in the surface oxic layers [17]. It has shown that the carbon-rich surface soil favored the development of α-*Proteobacteria* community [20], while the carbon-poor deeper layers need fungi to maintain the community functions because fungi have higher carbon assimilation efficiencies than bacteria [28].

### Genes Related to Arsenic Resistance

The arsenic-resistance genes were well represented in the five soil depths, with a total of 92 *arsC*/*B*/*A* gene sequences detected. Those gene sequences were retrieved from 58 bacterial genera, which reflected the high diversity of arsenic-resistant bacteria in this environment. Among the detected *ars* genes, 16 genes were shared across all five samples, and 29 genes were unique (detected at single depth) to a single sample level, with 19 of these genes unique to the surface sample (Fig. S1, 0-m).

Although soluble As concentrations were similar across the depths (Table 1), changes in the As-resistant microbial structures were detected (Fig. S1). Samples from deeper layers were clustered together, such as samples of 3-m and 4-m, while the surface sample separated very well from others (Fig. S1). In line with the whole community structure, the 3-m and 4-m samples shared the highest percentage of overlapped genes (62.5%). The correlation coefficient values showed similar trends, as the samples taken at 3-m and 4-m shared the highest correlation (Fig. S1). The upper layers processed significantly higher organic carbon contents (Table 1) that may play a critical role in As mobility, which appears to be an important factor in mitigating As toxicity [29]. The relative abundances of *ars* genes were higher in the deeper layer samples (data not shown), although the microbial diversities decreased sharply (lower detected *ars* genes) compared to the surface samples (Fig. S1). Our previous work showed that the As-contaminated level was the main driver in reducing the soil microbial diversity [7], while microorganisms could maintain the metabolic activity via changes in the structure towards a higher resistance community [11]. This leads to fewer dominant species that are better adapted to As contamination. A previous study also reported that subsurface microbial communities harbored a high adaptability in mercury-contaminated soils [30], which matches the results obtained here.

There was no significant correlation between the structure of *ars* genes and As concentrations (*p = *0.873), which appears contradictory to our previous work performed using adjacent surface soils [7]. There are several possible reasons that might explain this discrepancy. One is that a long-term and similar level As contamination across the depths (Table 1), resulting in minor effects on the selection of As resistant microbial communities. Accordingly, a great difference in the As-contaminated level led to significant effects on microbial community structures [7]. Another alternative explanation is the biogeochemical factors were as important as contaminants in shaping the microbial community [31], such as available carbon and spatial isolation [19]. Indeed, the C/N showed a marginally significant (*r = *0.742, *p = *0.069) effect on the structure of *ars* genes, which may reflect the integrated effects of the soil features that drive the differentiation of microbial composition [32].

### Functional Genes for Carbon Cycle

Carbon bioavailability is one of the primary determinants of soil microbial growth and activity [33]. The quantity and quality of the carbon substrate decline with soil depths (Table 1], which could strongly influence the structure of the microbial communities [18], [34]. Our results showed that the abundance of genes involved in carbon degradation significantly changed across the depths. Mantel tests revealed that C/N had a marginally significant correlation (*p = *0.073, *r = *0.84) with the relative abundance of carbon cycling genes. The deeper layer samples with low total carbon (TC) had a lower abundance of carbon degradation genes (Table 1, Fig. S2). It has been reported that substrate limited conditions supported slow nutrient cycles in which nutrients are conserved to improve soil carbon sequestration [50], thus, the lower abundance of carbon degradation genes in the carbon limited condition may preferably manage the community structure.

Rubisco is the predominant enzyme in the biosphere autotrophic bacteria that are known to be at the base of life because it provides the substrate for heterotrophic communities [35]. The TC and C/N decreased with depths, indicating that deeper soil horizons are more carbon limited, so the CO_2_ fixation should be more important to shape the microbial structure. A total of 78 Rubisco genes *rbcL* were detected, and approximately half of the genes (37/78) were unique in that they were only detected at a single depth; of these, 19, 3 and 15 unique genes were detected in 0-m, 2-m and 4-m soil samples, respectively (Fig. S3), indicating the significant effects of carbon limitation on *rbcL* gene’s distribution within soil depths. Furthermore, the distributions of the *rbcL* genes were significantly correlated with the soil TC concentration (*r* = 0.67, *p* = 0.071).

**Figure 3:**
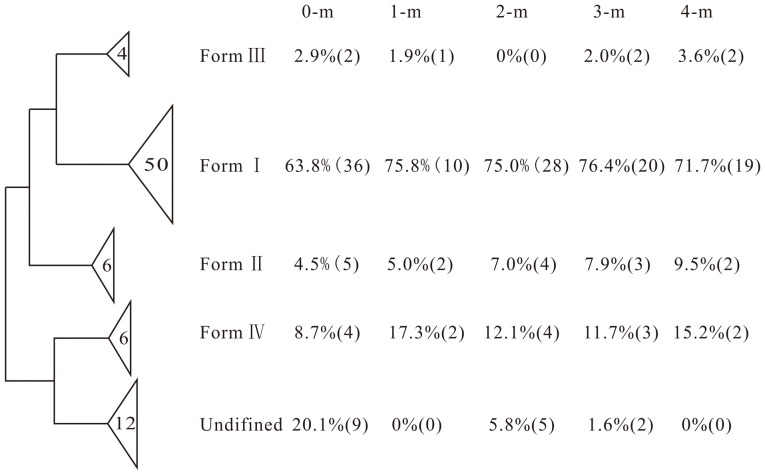
The distribution of *rbcL* genes. The width of each wedge is the number of *rbcL* sequences within each cluster. The percentages and numbers in each bracket are the signal proportions and detected gene numbers of each cluster within each depth, respectively.

Notably, several *rbcL*-like sequences retrieved from microbiota that are able to fix CO_2_ under aerobic conditions were only detected in the surface soils (0-m), such as *Xanthobacter autotrophicus* Py2 (gi 89362129) and *Alkalilimnicola ehrlichei* MLHE-1 (gi 114320324), while certain unique genes in the 4-m samples were retrieved from some anaerobic CO_2_ fixer, e.g., *Methanosaeta thermophila* PT (gi 116666356) and *Methanosarcina mazei* Go1 (gi 21227351) (Fig. S3). The distribution patterns of the unique genes were consistent with the vertical soil biogeochemical features, varied from aerobic, facultative anaerobic to anaerobic environments. Some species that belong to the *Proteobacteria* harbor multiple copies of Rubiscos, and these were detected across the soil depths, such as *Acidithiobacillus ferrooxidans*, *Synechococcus* sp. and *Burkholderia xenovorans* LB400 (Fig. S3). These microorganisms survive in a range of environments with a more flexible lifestyle that varies widely in the spatial and temporal variation of CO_2_ and O_2_ [3]. These facultative autotrophs processed diverse *rbcL* genes across the samples at different depths, allowing for growth on organic substrates as alternative carbon and energy sources [36], and are better adapted to variable environmental situations. In addition, higher relative abundances of Form I, II and IV *rbcL* genes were detected in 0-m samples [Fig. 3], which might be due to the carbon substrate limitation, since Rubisco Form I and II enzymes are directly involved in carbon metabolism [35], and their catabolic and anabolic pathways of carbon metabolism are inducible [37]. In contrast, the surface soils harbored an incredibly high relative abundance (20.1%) of undefined *rbcL* genes (Fig. 3), indicating that the surface soils exhibited a unique community and the metabolic properties were distinct from those at other soil depths.

### Functional Genes for Nitrogen Cycle

The nitrogen (N) dynamics is also a central issue in terrestrial ecosystems, while N transformations are mainly mediated by soil microorganisms. Although total nitrogen (TN) did not show significant correlations with the distribution of genes related to N mineralization, significant changes of each process were detected among the depths (Fig. S4). The signal densities of genes involved in denitrification were decreased across the soil profile and significantly correlated (*r* = 0.822, *p* = 0.032) with the NO_3_^−^ concentration. NO_3_^−^ is a N source in nutrient-limited environments, thus, lower microbial denitrification potential may favor the N sequestration and mitigate N limitation at deeper soils, although the linkage between gene abundances and system level process rates requires further study. Particularly, NO_3_^−^ could be used as an electron acceptor to generate energy or be coupled with arsenite oxidation to reduce As toxicity under anaerobic conditions [38].

It has been revealed that the ammonification rate had a linear correlation with microbial potential activity in soils [39]. The *ureC* genes that transform organic nitrogen to NH_4_^+^ were significantly decreased in the deeper layer samples (Fig. S4), indicating lower microbial activities at those soil depths. Notably, the variable pattern of genes involved in dissimilatory N reduction was distinct from other N cycle processes. Specifically, the abundance of *napA* was not significantly changed among the five depths, while *nrfA* was even higher at the deeper layers (Fig. S4). It is highly possible that the bacterial reduction of nitrite to ammonia is a survival strategy during oxygen starvation in deeper layers [40].

**Figure 4:**
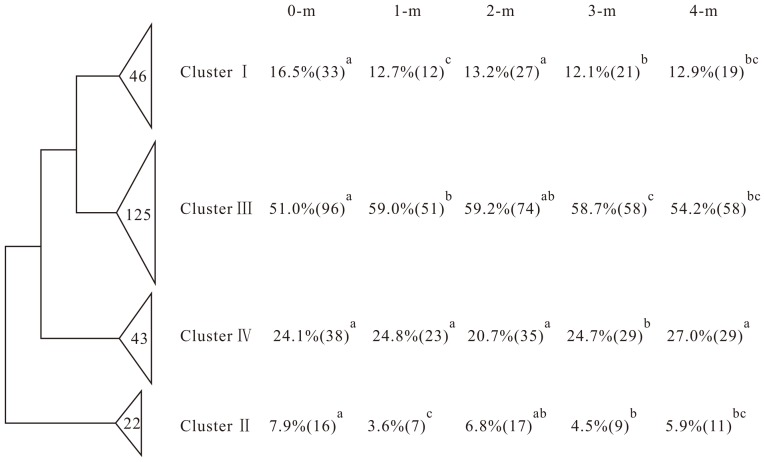
Maximum-likelihood phylogenetic tree of the 236 different *nifH* gene sequences obtained from GeoChip 3.0 analysis. The width of each wedge is the number of *nifH* sequences within each cluster. The percentages and numbers in each bracket are the signal proportions and detected gene numbers of each cluster within each depth, respectively. The significant differences of gene abundance were analyzed by one-way ANOVA.

The biological N fixation process provides the major N source, and thus *nifH* genes have been widely used to detect N_2_ fixers [41], [42]. The surface sample harbored a higher diversity of *nifH* genes compared to the deeper layer samples. Among the *nifH* genes, cluster III genes were predominant (>50%) in all samples (Fig. 4), as this cluster contained the largest and most divergent phylogenetic groups, some of which may be paralogues arising from a duplication of *nifH* during early evolution [43]. Gene sequences of Cluster III were retrieved from anaerobic or microaerophilic habitants, and their relative abundances were increased in subsurface soils, albeit that other clusters did not show such a trend (Fig. 4). The increased soil depths were oxygen limited with air-filled pore space strongly related to depth [44], [45], which may select for anaerobic N_2_-fixers and favor N fixation. Sulfate-reducing bacteria (SRB) are known to be N_2_-fixers and harbor nitrogenase genes, and these were also detected in Cluster III, such as *Desulfovibrio*, *Desulfotomaculum* and *Syntrophobacter* (Data not shown). Meanwhile, a relatively high diversity of Cluster I *nifH* genes was observed (Fig. 4), which was consistent with the widespread distribution of Proteobacteria. Interestingly, the *nifH* genes within Cluster IV (contains divergent archaeal *nifH* sequences) showed a subdominant relative abundance (Fig. 4). Some archaea that have been isolated from arsenic-contaminated sediments, such as *Desulfitobacterium*, are important for arsenic metabolism and transformation [46]. The high abundances of detected archaeal *nifH* sequences had not been discerned previously.

There were a significant correlation between the distribution of *nifH* genes and C/N (*r = *0.751, *p = *0.024). Previous studies have shown that N fixation activities are positively correlated with C/N in the *Cyanobacteria* [47] and that C/N can be a major driving force for a shift in soil bacterial communities [48]. Though the presence of *nifH* genes in the habitat may not be directly linked to the process catalyzed by the expressed protein; it can provide a biomarker for the change and basis of microbial community structures.

### Functional Gene for Phosphorus Cycle

Phosphorus (P) is an important nutrient element that is often limited in soil ecosystems, while microbial mineralization is a major pathway for producing bioavailable P [49]. P and As compete at the sorption sites due to their physicochemical similarity [50]. Thus, microbial phosphorus-utilizing genes have an important effect on As mobility and transformation to groundwater.

A total of 129 phosphorus-utilizing genes [including polyphosphate kinase (*ppk*), exopolyphosphatase (*ppx*) and phytase genes] were detected, and most of these (108) were observed in the surface sample (Fig. S4). The distribution and abundance of the P cycling genes were significantly (*r* = 0.946, *p* = 0.018) correlated with soil P concentrations. Among the detected phosphorus-utilizing genes, 26 genes were shared across the soil depths and 57 genes were unique. Although most unique genes [51] were detected in the surface samples, several unique genes were detected in the deeper soil samples with high gene abundances (Fig. S5, red labeled). Our previous study has showed an intense correlation between microbial As and P metabolism at surface soils [7], while the variation of microbial P metabolism activities under aerobic and anaerobic conditions was unknown. Furthermore, the signal intensities of P cycling genes were significantly decreased in subsurface soils relative to those in the surface soils (data not shown), indicating a lower microbial P metabolism activity at the deeper layers.

### Functional Genes Involved in Energy Process

**Figure 5:**
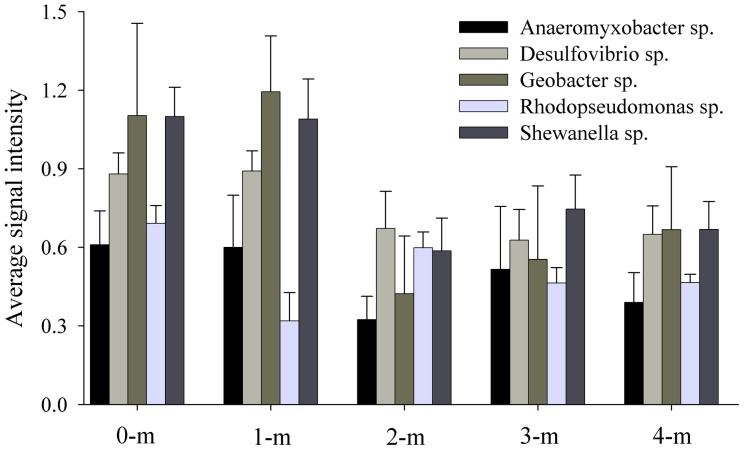
Average signal intensity of cytochrome *c* genes detected from different microorganisms. Error bars indicate ± 1 SE (*N = *6).

Cytochrome *c* oxidase (Cytc) is an essential enzyme that provides a critical function in cellular respiration and energy generation. Gene sequences retrieved from bacteria that represent α*-Proteobacteria* (*Rhodopseudomonas*), γ*-Proteobacteria* (*Shewanella* and *Pseudomonas*) and δ-*Proteobacteria* (*Anaeromyxobacter*, *Desulfovibrio* and *Geobacter*) were detected across the soil depths (Fig. 5). Notably, most of these microorganisms are arsenic resistant and capable of arsenate dissimilatory respiration [13]. However, significant changes were observed in the average intensities of these microorganisms based on the detected *cytC* genes (Fig. 5). The average signal intensities of these microorganisms from upper layers (0-m and 1-m) were much higher than those from deeper layers with higher moisture (2–4 -m). The limited electron acceptors, such as O_2_ or NO_3_^−^, could be crucial factors in controlling the respiration rate in subsurface of soils, resulting in the differentiation of community structure and activity across the vertical profile. Recent studies have used additional electron donors or removed dissolved oxygen to stimulate U(VI) reduction/immobilization [52] and/or improve the U(VI) bioremediation rate [53]. Conversely, the electron acceptor could be manipulated during As bioremediation, especially under anaerobic conditions because the oxidation of arsenite to arsenate largely reduce As toxicity and mobility [51].

### Relationship between Microbial Community Structure and Environmental Factors

The top four (P, NO_3_^−^, C/N and As) geochemical variables were identified via forward selection by canonical correspondence analysis (CCA) to correlate them with the community structure. The arrow length of As was short and almost overlapped with that of C/N. To reduce the variance inflation factors, As was removed, and the CCA was repeated. The specified CCA biplot revealed that the variation was marginally significant (*r* = 0.694, *p = *0.074) with the combination of C/N, NO_3_^−^ and P (Data not shown). This suggests that microbial communities had been adapted to arsenic stress after a long-term exposure to arsenic.

**Figure 6:**
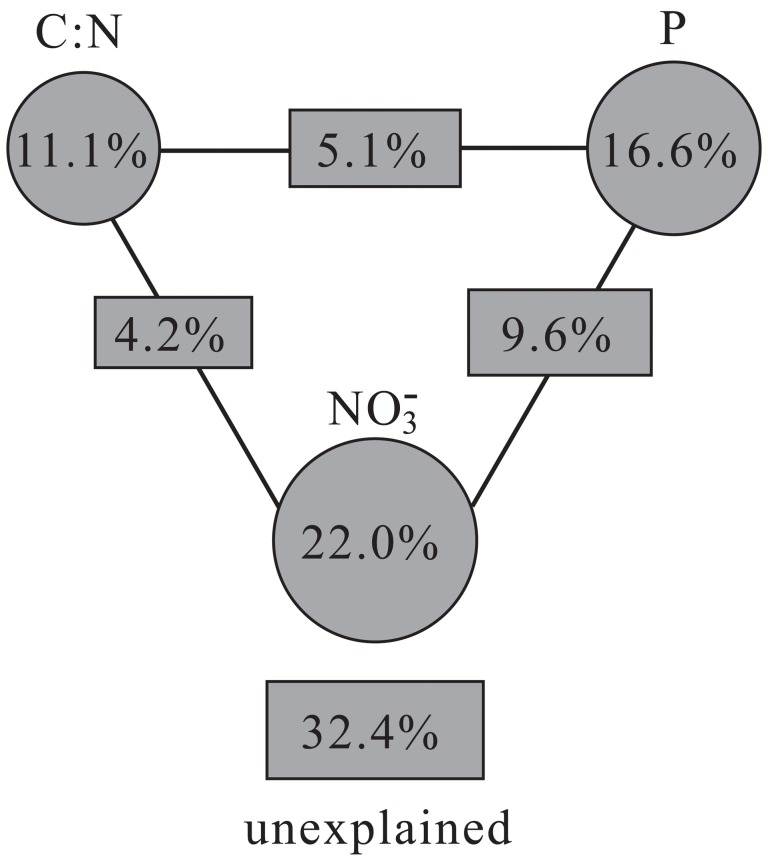
Variation partitioning analysis of microbial diversity variance among important geochemical variables, P, NO_3_^−^ **, and C/N, and their interactions.**

Variation portioning analysis was preformed to better understand how much of each variable influenced the functional community structure (Fig. 6). Three variables, P, NO_3_^−^ and C/N, explained a large portion of the observed variation, leaving 32.4% of the variation unexplained. The C/N alone explained 11.1% (*p* = 0.079), P attributed to 16.6% (*p* = 0.068), and NO_3_^−^ explained the largest amount of variation, 22.0% (*p* = 0.053). The interactions between C/N and P, C/N and NO_3_^−^, P and NO_3_^−^ accounted for 5.1%, 4.2% and 9.6% contributions, respectively (Fig. 6). These results indicated that P, NO_3_^−^ and C/N influenced the microbial community structures to a large extent. Nitrate can serve as electron acceptor to oxidize As(III) under anaerobic conditions [13], [33]. This process is largely reduces As toxicity and provides energy to heterotrophic habitants [33], and thus NO_3_^−^ is the predominant factor triggering the shift in microbial community. The unexplained amount of variation in this study (32.4%) was higher (24.9%) than the As-contaminated rhizosphere of *P. vittata* in the same area [7], indicating that the distribution mechanism of spatial isolation was more complex, while it was similar (35.9%) to a Tc/U-contaminated groundwater survey [54]. Additionally, unexplained variation may be the result of other geochemical factors, such as oxygen vertical gradient, which exert effects on the microbial community structure [32].

### Conclusion

Understanding the forces governing microbial community assembly is critical in microbial ecology, especially as it relates to arsenic bioremediation. In this study, the robustness of GeoChip and BioLog analyses were integrated to examine the microbial community structures, key functional genes and metabolic potential along a vertical soil profile with long-term arsenic contamination. The microbial metabolic potential substantially decreased with soil depth. Significant changes of key functional genes related to As resistance, C and N cycling, P utilization and the energy process were observed, which were closely correlated with spatial isolation or geochemical features, such as C/N. Significant differences in microbial community structures were detected based on *rbcL* and *nifH* genes as biomarkers, which consistent with the physical pattern from aerobic to anaerobic conditions along this vertical profile. Although a number of factors may concomitantly shape the differentiation of soil microbial communities, our results revealed that the combination of P, NO_3_^−^ and C/N showed the highest correlation with the microbial community structure, while the pivotal effects of a long-term (16 years) arsenic downward infiltration was not as predictive as expected.

Here, we extend the ideas that spatial isolation and vertical heterogeneity (e.g., nutrient and electron acceptor availability) were the definitive factors in controlling the microbial community structures and activities although long-term As contamination would have minor effect on the microbial community. From a practical standpoint, understanding the key factors shaping vertical soil microbial communities and spatial distribution patterns is essential to stimulate and maintain desired populations to achieve As bioremediation goals in the future.

## Supporting Information

Figure S1 Hierarchical cluster analysis of arsenic resistant genes. Samples grouped with soil depths.
(TIF)

Figure S2 The abundance of detected key genes involved in carbon degradation. All data are presented as the mean ± SE (standard error).
(TIF)

Figure S3 Maximum-likelihood phylogenetic tree of the 78 different Rubisco gene sequences obtained from GeoChip 3.0, showing the phylogenetic relationship among the five *rbcL* clusters. The genes detected are shown in bold with the gene ID in the front. The green, blue and red font colors represent unique genes in the 0-m, 2-m and 4-m samples, respectively.
(TIF)

Figure S4 The abundance of detected key genes related to nitrogen cycle. (A) N2 fixation, *nifH* encoding nitrogenase; (B) Nitrification, *amoA* encoding ammonia monooxygenase; (C) Denitrification, including *narG* for nitrate reductase, *nirS* and *nirK* for nitrite reductase, *norB* for nitric oxide reductase and *nosZ* for nitrous oxide reductase; (D) Dissimilatory N reduction to ammonium, including *napA* for nitrate reductase and *nrfA* for c-type cytochrome *nitrite* reductase; (E). Ammonification, including *gdh* for glutamate dehydrogenase and *ureC* encoding urease; (F) Assimilatory N reduction, *nasA* encoding nitrate reductase. All data are presented as the mean ± SE.
(TIF)

Figure S5 Hierarchical cluster analysis of phosphorus-utilizing genes among soil samples of different depths (shared genes among the depths are not shown). The red labeled genes are the unique genes in the 1-m or 2-m samples with high abundance.
(TIF)

Table S1 Detected gene numbers and diversities (average values ± SE at each depth) and of the microbial community.
(DOC)
