# Supplementary figures and images for: Assessing the Microbial Community and Functional Genes in a Vertical Soil Profile with Long-Term Arsenic Contamination
Source: PLoS One. 2012 Nov 30;7(11):e50507. doi: 10.1371/journal.pone.0050507 (PMC3511582; doi:10.1371/journal.pone.0050507)

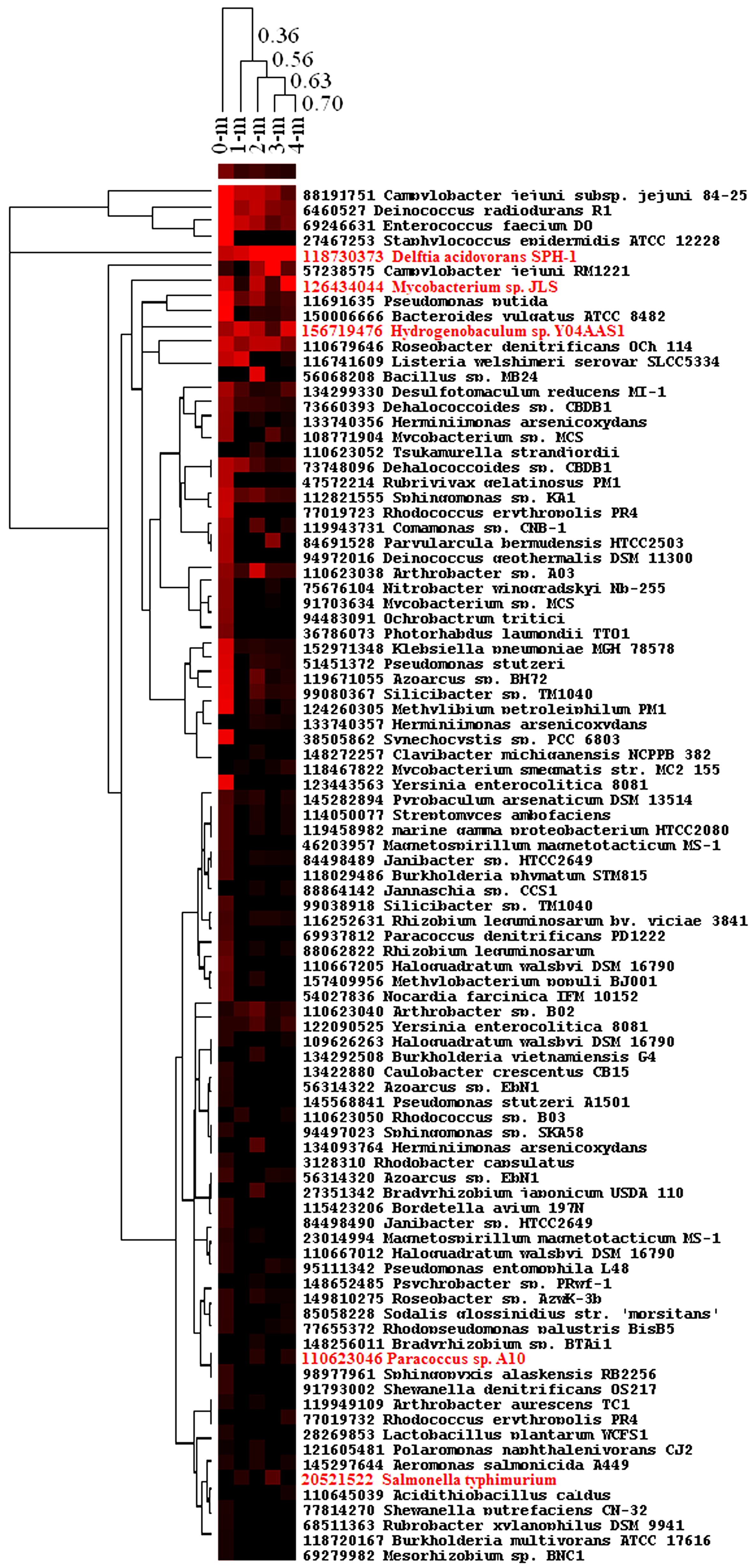

Supplement: Figure S1 — Hierarchical cluster analysis of arsenic resistant genes. Samples grouped with soil depths. (TIF) [file pone.0050507.s001.tif]

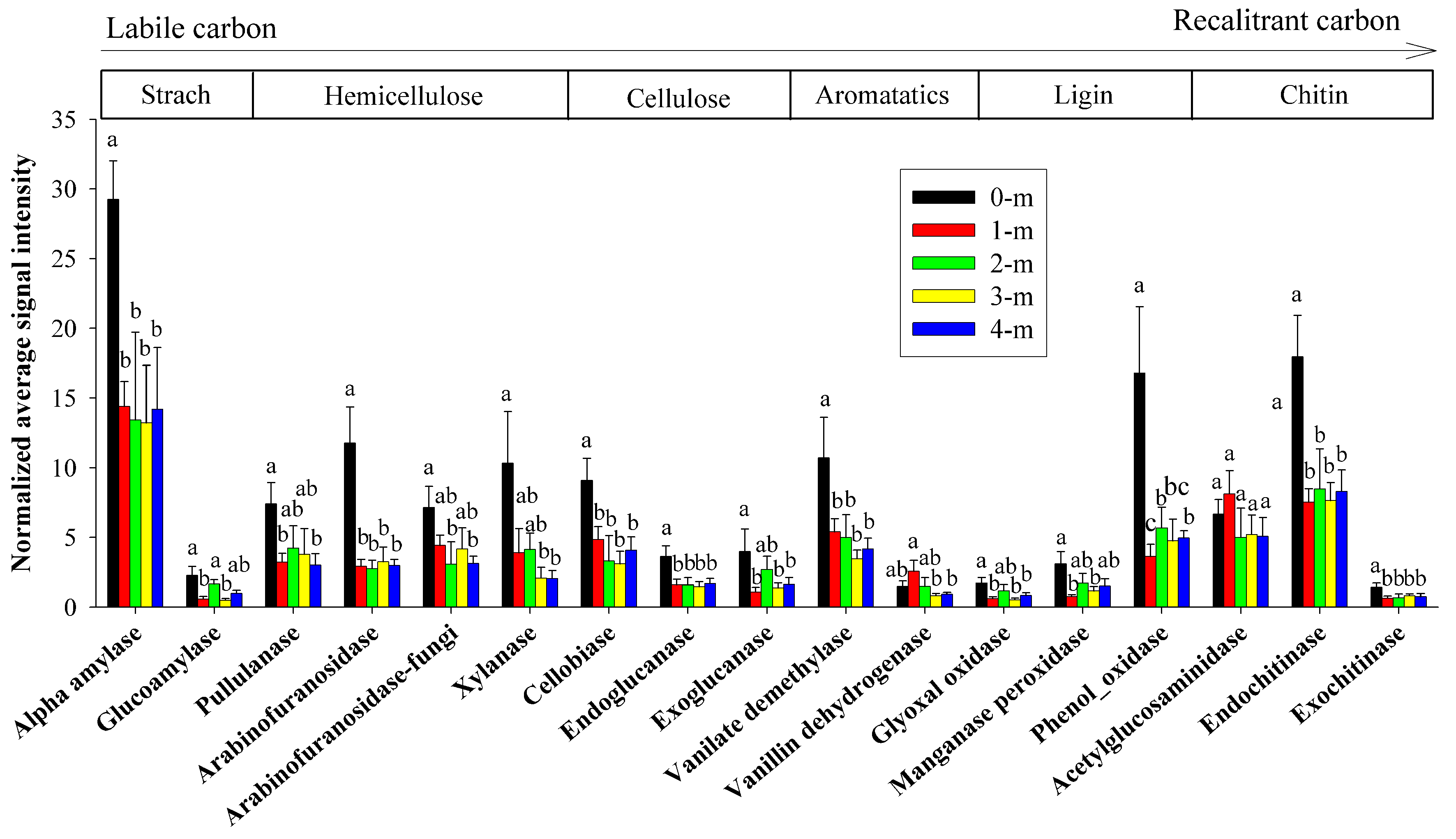

Supplement: Figure S2 — The abundance of detected key genes involved in carbon degradation. All data are presented as the mean ± SE (standard error). (TIF) [file pone.0050507.s002.tif]

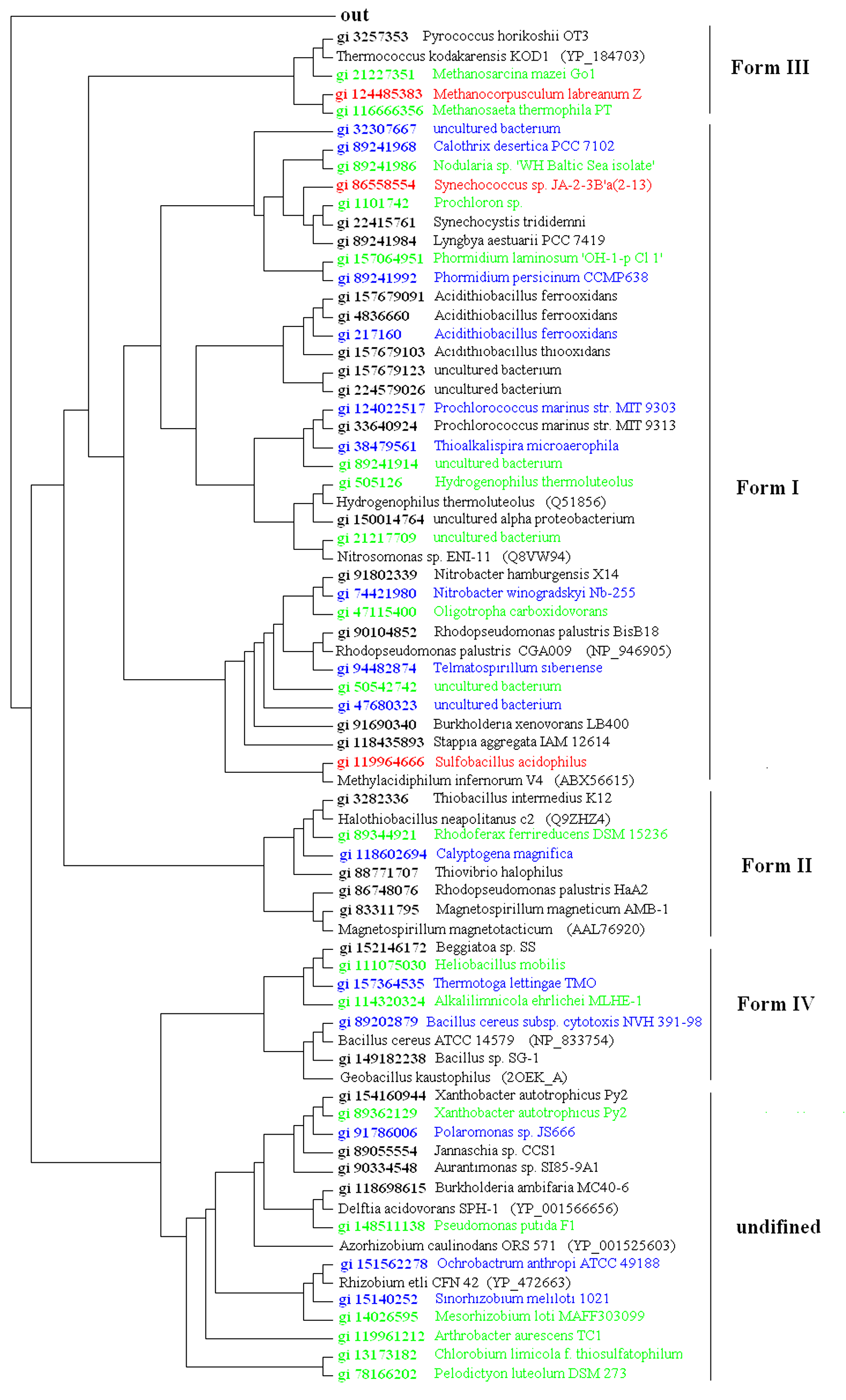

Supplement: Figure S3 — Maximum-likelihood phylogenetic tree of the 78 different Rubisco gene sequences obtained from GeoChip 3.0, showing the phylogenetic relationship among the five rbcL clusters. The genes detected are shown in bold with the gene ID in the front. The green, blue and red font colors represent unique genes in the 0-m, 2-m and 4-m samples, respectively. (TIF) [file pone.0050507.s003.tif]

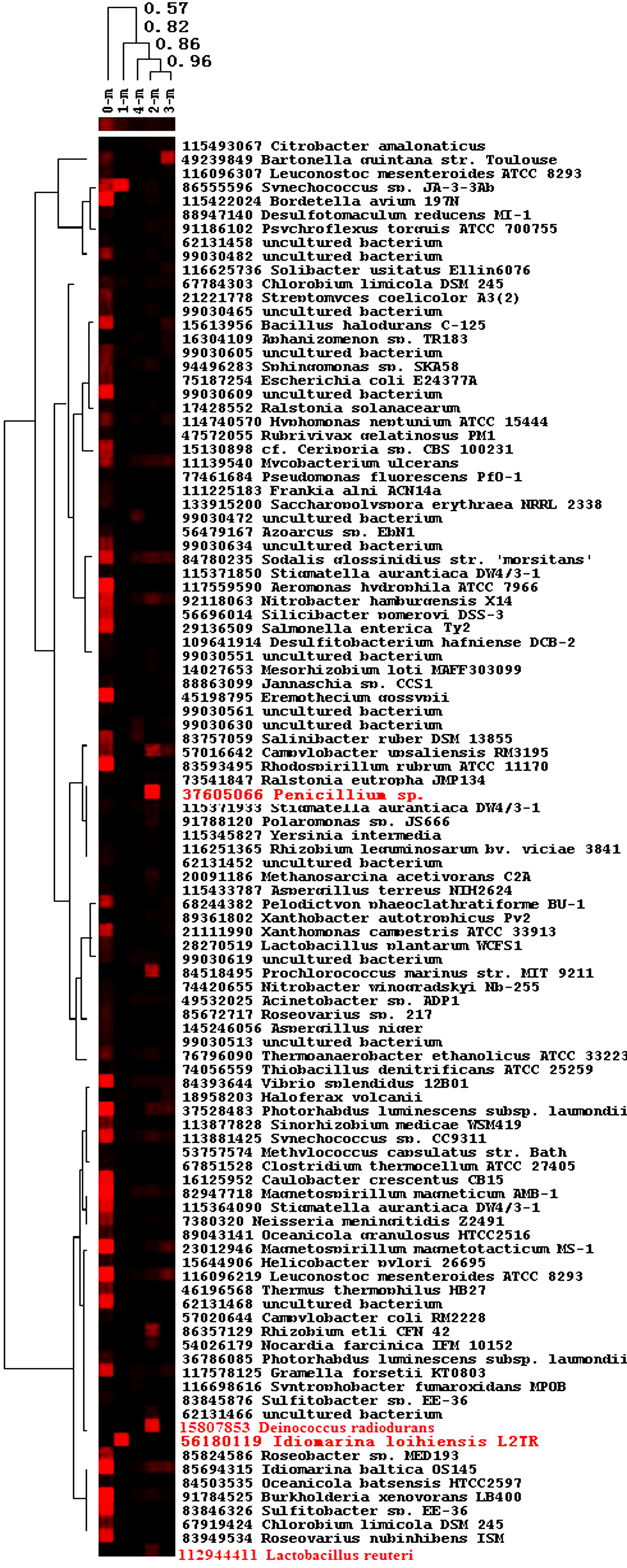

Supplement: Figure S5 — Hierarchical cluster analysis of phosphorus-utilizing genes among soil samples of different depths (shared genes among the depths are not shown). The red labeled genes are the unique genes in the 1-m or 2-m samples with high abundance. (TIF) [file pone.0050507.s005.tif]
