# Supplementary material for: Assessing the Microbial Community and Functional Genes in a Vertical Soil Profile with Long-Term Arsenic Contamination
Source: PLoS One. 2012 Nov 30;7(11):e50507. doi: 10.1371/journal.pone.0050507 (PMC3511582; doi:10.1371/journal.pone.0050507)
Supplement: Table S1 — Detected gene numbers and diversities (average values ± SE at each depth) and of the microbial community. (DOC) [file pone.0050507.s006.doc]

**Table S1.** Detected gene numbers and diversities (average values ± standard error at each depth) and of the microbial community

| Index | 0-m | 1-m | 2-m | 3-m | 4-m |
| --- | --- | --- | --- | --- | --- |
| Gene number | 2489±392^a^ | 882±225^c^ | 1522±412^b^ | 1230±229^bc^ | 1464±122^bc^ |
| Simpson Index (*1/D*) | 1433.0±272^a^ | 485.5±60^c^ | 840.0±199^b^ | 659.0±42^bc^ | 835.5±81^bc^ |
| Shannon Index (*H'*) | 7.47±0.39^a^ | 6.48±0.25^b^ | 6.86±0.70^ab^ | 6.82±0.19^ab^ | 7.03±0.21^ab^ |

One-way ANOVA procedures with Tukey test to detect the significant differences of diversity among samples at different depths.
